# Neurological presentation of Whipple's disease after long-term antibiotic treatment: a case report

**DOI:** 10.1186/1752-1947-2-191

**Published:** 2008-06-03

**Authors:** Felix Gundling, Henning Wittenburg, Andrea Tannapfel, Joachim Mossner

**Affiliations:** 1 Second Department of Medicine, Bogenhausen Hospital, Academic Teaching Hospital, Technical University of Munich, Munich, Germany; 2 Second Department of Medicine, University of Leipzig, Leipzig, Germany; 3 Department for Pathology, University of Leipzig, Leipzig, Germany

## Abstract

**Introduction:**

Whipple's disease is a rare systemic infectious disorder caused by *Tropheryma whipplei*.

**Case presentation:**

We report a 68-year-old male with Whipple's disease of the central nervous system following long-term antibiotic therapy and many years after the initial clinical onset.

**Conclusion:**

The combination of trimethoprim and sulphamethoxazole does not prevent or cure involvement of the central nervous system in all patients with Whipple's disease. If relapse of the central nervous system occurs treatment with meropenem might be a useful alternative.

## Introduction

Whipple's disease (WD) is a rare condition caused by infection with Gram-positive bacilli (*Tropheryma whipplei*) characterised by fever, diarrhoea, arthropathy, apathy and cranial nerve lesions, among other symptoms [1]. WD of the central nervous system (CNS) can be observed even after long-term antibiotic therapy and many years after clinical onset [2,3].

## Case presentation

We evaluated a 68-year-old man, who presented with drowsiness, progressive memory loss, dysarthria and muscular weakness, which was observed 6 months previously. On physical examination, the patient was somnolent and not adequately orientated. Neurological examination revealed complete paresis of the oculomotor nerve and incomplete paresis of the abducent nerves, mild weakness of bilateral lower limbs and a left Babinski sign. The patient's past medical history was remarkable for WD. In 1992, the patient experienced weight loss and diarrhoea resulting in a severe malabsorption syndrome combined with diffuse arthropathy. WD was diagnosed upon positive staining of macrophages of the duodenal mucosa for peroxidase acid-Schiff (PAS) reagent. Long-term antibiotic therapy was carried out with doxycycline (2 × 100 mg), which was changed later to trimethoprim-sulphamethoxazole (2 × 160/800 mg) because of photoallergic reactions. The symptoms improved rapidly accompanied with gradual reconstitution of the villous architecture of the small intestine. Antibiotic therapy was discontinued in 1995 after 36 months of treatment.

The patient's past medical history further revealed a thoracotomy in 1987 for complete removal of a bronchial carcinoid of the left bronchus. No chemotherapy was administered and there was no sign of relapse or metastasis in follow-up examinations.

**Figure 1:**
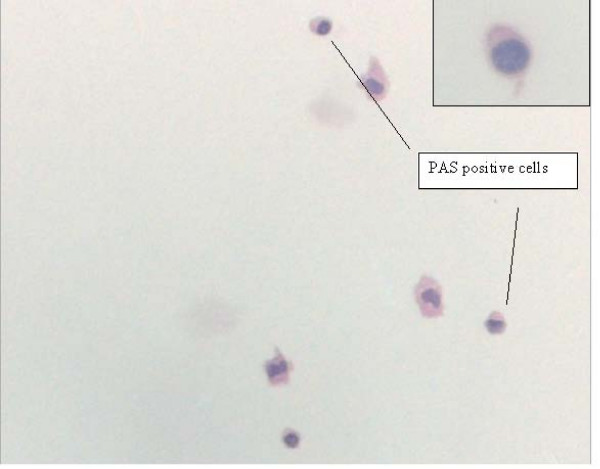
**Peroxidase acid-Schiff-positive cells obtained from cerebrospinal fluid**. Original magnification ×20.

He represented after an interval of 12 years. Laboratory tests on admission to the hospital displayed an increased C-reactive protein level (18.6 mg/l: normal range -5) and an elevated leukocyte count (11.3 × 10^9 ^leucocytes/l: normal range 4 to 9/nl). All other routine laboratory results were within normal limits. Lumbar puncture revealed a mildly elevated protein level (510 mg/l: normal range 150 to 450 mg/l) and a modest pleocytosis (6 Mpt/l). Cerebrospinal fluid (CSF) glucose was within normal limits. Examinations of cell pellets obtained by cytocentrifugation of CSF samples were positive for PAS staining (Figure 1). Polymerase chain reaction (PCR) testing of the CSF for the causative agent of WD was positive resulting in strong presumptive evidence of CNS infection by *Tropheryma whipplei*. Brain computed tomography (CT) displayed diffuse hypodense lesions in subependymal grey matter. An endoscopy of the upper gastrointestinal tract revealed erosive duodenitis with no histopathological characteristics for WD. Brain biopsy was not performed. Intravenous antibiotic treatment with meropenem (2 × 1 g) for 2 weeks was carried out followed by treatment with oral trimethoprim-sulphamethoxazole (2 × 160/800 mg) according to the study protocol of the first prospective antibiotic trial in WD ('Study for the Initial treatment of Morbus Whipple' (SIMW)) [1]. While receiving antibiotic therapy, the patient's vigilance improved. However, his memory ability remained impaired in the follow-up examination indicating possible irreversible damage to the brain caused by WD disease of the CNS.

## Discussion

WD, named after the American pathologist George H Whipple, who described this disease as 'intestinal lipodystrophy' in 1907, is a rare systemic infectious disorder with an estimated incidence of 0.4 per 1,000,000 a year [1]. The disease is characterised by intestinal involvement but includes a large number of other organs, especially the lymphatic system, heart and CNS [4]. The most common clinical features include weight loss and abdominal pain (approximate frequency 80% to 90% and 50% to 90%, respectively) [1]. The two major problems of WD are CNS disease and relapsing infection. CNS manifestations have a frequency of up to 15% and can occur in rare instances with little or no gastrointestinal involvement [1]. CNS involvement may be silent and its frequency, therefore, is underestimated [2]. Patients may present in a variety of ways, including cognitive impairment, psychiatric manifestations, gaze palsies, upper motor neurone signs and hypothalamic dysfunction. Further neurological symptoms include disorders of eye movement, for example, ophthalmoplegia and nystagmus, complex cranial-nerve manifestations and myoclonus [1,5]. Some authors described an oculomasticatory myorhythmia as pathognomonic for cerebral WD [6]. The triad of dementia, supranuclear ophthalmoplegia and myoclonus is highly suggestive of WD. In contrast, pure oculomotor palsies are rare [7].

The CSF, typically showing inflammatory cell responses with occasionally PAS-positive macrophages, should be analysed before treatment and during follow-up [3]. PCR may also be used. Primary WD of the brain may be diagnosed by molecular biological techniques such as PCR [4,8,9]. PCR can also be performed on the CSF. Other examinations include CT or magnetic resonance imaging of the brain, which may display atrophic alterations, mass lesions and hydrocephalus. This explains the sometimes focal neurology secondary to solitary mass lesions, for example, generalised tonic/clonic seizures or symptoms resembling a stroke syndrome [2,9]. In patients with WD who have predominant digestive involvement, intestinal biopsies for histology should be indicated first and, if negative, a bacterial PCR determination should be the next option [1]. Although the molecular PCR assessment of cerebral biopsies has the highest diagnostic yield in neurological WD, its associated morbidity means that analyses of intestinal samples are more appropriate. The use of PCR techniques, however, remains limited to specialized research laboratories.

Treatment of WD is still empirical as the final results of the first prospective trial initiative SIMW are not yet available. In this trial, two highly active antibiotics (ceftriaxone and meropenem) known to penetrate the blood-brain barrier administered for 14 days followed by 12 months maintenance treatment with cotrimoxazole were compared. Currently, recommended antibiotic therapies include an initial 2-week course of intravenous cephalosporins (for example, ceftriaxone) or meropenem for 2 weeks followed by 1-year oral trimethoprim-sulphamethoxazole [1]. As described before, clinical relapses such as in our patient with recurrent CNS manifestation may occur even after long-term oral antibiotic treatment with trimethoprim-sulphamethoxazole if the causative agent was not eradicated timely from the CNS [10]. Patients treated primarily with tetracycline show a high relapse rate of 35%, especially CNS relapse. One case presentation describes a successful treatment with recombinant interferon gamma (INF-gamma) together with antimicrobial therapy, which led to clearance of infection in a chronically relapsing patient [11].

## Conclusion

The incidence of WD is low despite the ubiquitous presence of *T. whipplei *in the environment [12]. Furthermore, sites of organ infection show a remarkable lack of inflammatory response to the bacterium. Therefore, it has been suggested that host factors indicated by immune deficiencies are responsible for the development of WD. Recent data demonstrate a persistent defect of the cellular immune response with decreased serum concentrations of interleukin (IL-12), INF-gamma and tumour necrosis factor (TNF)-alpha [12]. Other results indicate an association between immunosuppressive therapy and symptomatic onset in WD and thus support the concept that immunological factors play a role in disease pathogenesis [13]. Some authors, as we do, have described an association between malignant tumour occurrence and an increased relapse risk of WD in oncological patients. Some reports have described malignant non-Hodgkin lymphoma a few years after diagnosis of WD. In one case presentation, an intramucosal gastric adenocarcinoma was diagnosed 8 years after initial manifestations of WD [14,15]. To date, it is unclear whether malignant tumours increase the incidence and relapse risk of WD. This question must be answered by further epidemiological studies.

## Abbreviations

CNS: central nervous system; CSF: cerebrospinal fluid; CT: computed tomography; INF-gamma: interferon gamma; PAS: peroxidase acid-Schiff; PCR: polymerase chain reaction; SIMW: Study for the Initial treatment of Morbus Whipple; TNF-alpha: tumour necrosis factor-alpha; WD: Whipple's disease.

## Competing interests

The authors declare that they have no competing interests.

## Authors' contributions

FG carried out the clinical examination and drafted the manuscript, HW approved the version to be published, AT performed the histological examination of cerebral liquor, JM gave final approval of the version to be published. All authors read and approved the final manuscript.

## Consent

Written informed consent was obtained from the patient for publication of this case report and any accompanying images. A copy of the written consent is available for review by the Editor-in-Chief of this journal.
